# What role do traditional pharmacology textbooks play in medical students’ education and exam preparation?

**DOI:** 10.1007/s00210-025-04459-3

**Published:** 2025-09-01

**Authors:** Delany Manotheepan, Roland Seifert

**Affiliations:** Institute of Pharmacology, Hannover Medical School, D-30625 Hannover, Germany

**Keywords:** Pharmacology textbooks, Digital learning platforms, AMBOSS, Via Medici, Medical studies, Exam preparation, Teaching materials, Learning behavior, Online survey, Didactic preparation, Interactivity, Dynamic learning formats, Exam-relevant content, Textbook development

## Abstract

Textbooks have traditionally been a central component of medical education. However, with the rise of digital learning platforms such as AMBOSS and Via Medici, questions have arisen regarding the current relevance of traditional pharmacology textbooks in the study habits of medical students. This study aims to assess the extent to which textbooks are used for exam preparation, identify the most frequently used titles, and explore the factors influencing a shift toward alternative learning methods. An anonymous nationwide online survey was conducted among medical students in Germany from the fifth semester onward. The frequency of textbook usage was assessed quantitatively, while popular textbook choices and motivations for using digital resources were analyzed qualitatively. The findings indicate that while traditional textbooks are still in use, their importance is decreasing relative to digital learning platforms. Nineteen percent of participants reported using textbooks as a primary learning resource, while around 30% primarily relied on digital platforms. Frequently cited reasons for reduced textbook use included the additional time investment required, limited interactivity, and lack of clinical applicability. Moreover, many students expressed a preference for more concise and comprehensible content, including mnemonics, as well as more effective visual representation through tables and illustrations. The present findings underscore the necessity for pharmacology textbooks to evolve with students’ learning preferences. At the same time, there is potential for improvement, particularly in terms of instructional design and the focused delivery of exam-relevant material. This study provides insights for the future development of teaching materials in medical education.

**Supplementary Information:**

The online version contains supplementary material available at 10.1007/s00210-025-04459-3.

## Introduction

For decades, textbooks have been an indispensable source of knowledge in medical education. Pharmacology, in particular, is known for its content complexity, making structured resources essential. Textbooks play a central role by presenting pharmacological principles, mechanisms of action, and therapeutic applications. In recent years, however, digital learning platforms such as AMBOSS have become increasingly important. These platforms allow for flexible and interactive learning and are often tailored to exam-relevant content. As a result, the way students engage with traditional textbooks has shifted.

A study by Riedel et al. (2019) demonstrated that medical students are increasingly relying on digital learning platforms, particularly for exam preparation. One major advantage of these platforms is their ability to stay up-to-date and adapt to evolving examination formats.

Keeping content up to date is also a crucial factor when evaluating the continued relevance of traditional textbooks. An analysis by Misera and Seifert (2024) revealed that pharmacological textbooks sometimes contain outdated information. Using the example of reserpine, the authors illustrated how different textbooks presented this drug across various editions. While some textbooks reflected their limited clinical relevance, others still included comprehensive descriptions of their supposed (but non-existent) clinical use. This highlights the need for regular updates to ensure consistency with current clinical guidelines and evidence-based practice.

Nevertheless, textbooks remain valuable, especially for an in-depth understanding of pharmacological principles. The decision to use textbooks versus digital learning platforms depends on multiple factors, including content, quality, relevance to current practice, and individual learning preferences.

A study by Egle et al. (2015) found that medical students and junior doctors prefer different sources of information depending on the situation. While students still use traditional textbooks and exam preparation materials, both groups tend to rely on digital resources for clinically oriented questions. Platforms such as UpToDate, Google, Medscape, Wikipedia, and Epocrates were among the most frequently used. These sources were evaluated using clinical questions based on realistic clinical situations such as ward rounds, written exams, and case discussions. UpToDate and Epocrates provided correct answers in 47% of cases, while Wikipedia had the lowest accuracy at 26%. None of the electronic sources was able to reliably answer more than half of the clinical questions. Despite these limitations, digital resources are widely favored due to their ease of access. Traditional textbooks, however, continue to play an important role, particularly when studying topics beyond those strictly required for exams.

The aim of this study is to assess how pharmacology textbooks are currently used by medical students in Germany and how they can be better aligned with students’ evolving learning needs. The following research questions are addressed:

- What factors influence students’ decisions to use traditional textbooks versus digital learning platforms?
- How can textbooks be adapted to meet the needs of today’s medical students?

To answer the questions, we conducted an anonymous online survey among medical students in Germany.

### Methodology

**Fig. 1:**
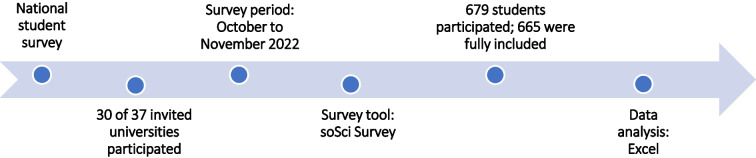
**Data collection process.** Process of data collection from the creation of the survey to the final data analysis

An anonymous online survey was conducted across Germany to assess the use of and students’ perspectives on pharmacology textbooks (see Fig. 1). The target group included students from the fifth semester up to the final year of clinical training (sixth year of study).

#### Study design and implementation

The survey took place between October and November 2022 and targeted students enrolled at 37 public universities across Germany. In total, students from 30 universities participated. Data collection was carried out using the online platform SoSci Survey, which included a range of question formats such as single- and multiple-choice questions, open-ended responses, and dropdown menus.

Textbooks were specifically selected based on their primary use at the Hannover Medical School (MHH). Special attention was given to including those commonly used in preparation for the state medical examination (Staatsexamen). The selection was primarily based on the editions of textbooks available at the university library of MHH during the time of the survey. It should be acknowledged that the editions students used may have differed based on individual access and preferences.

## Questionnaire composition

The questionnaire consisted of eleven closed and open-ended questions addressing the following aspects:

1. What year of study are you currently in? (single choice)
2. Which university do you attend? (dropdown menu)
3. What importance do you assign to pharmacology in your studies or future professional life? (single choice)
4. How would you evaluate the pharmacology teaching at your university? (single choice)
5. How did you prepare for the pharmacology exam? (multiple choice and open text)
6. How did you prepare or how would you like to prepare for pharmacology as part of the second state medical examination? (multiple choice and open text)
7. How thoroughly have you studied pharmacology? (single choice)
8. Which textbooks did you use for exam preparation? (multiple choice with open-text option)
9. Have you used English-language literature? If yes, which textbook? (single choice with open-text option)
10. What factors would encourage you to study more with pharmacology textbooks? (multiple choice with open-text option)
11. How well do you feel your studies in pharmacology have prepared you for your professional career? (single choice)

### Sample characteristics and representativeness

The target group included medical students from the fifth semester onward. This group was considered appropriate, as they had already taken part in pharmacology courses and could provide informed responses regarding the use of textbooks and digital learning resources. Furthermore, many had already completed the pharmacology exam or were preparing for it.

The sample comprised students from various academic years and institutions. Of the 679 respondents, 665 completed the questionnaire in full. To evaluate the representativeness of the sample, we analyzed participant distribution by university and year of study. The results showed a broad representation of institutions, although some universities were overrepresented.

In addition to reporting the total number of participants, particular attention is given to MHH as the institution with the highest number of respondents, to all universities excluding MHH, and to the University of Leipzig, which had the second-highest number of participants.

#### Data collection and analysis

The data were analyzed using Microsoft Excel and visualized using appropriate graphical techniques. Pie charts and bar graphs were used to present the results.

## Results and discussion

### Study survey

The results of the study survey were analyzed with consideration of the total number of participants. Due to the high participant numbers, special focus was placed on the responses from MHH, with 167 participants, and Leipzig University, with 55 participants.

### Distribution of participants by academic year

**Fig. 2:**
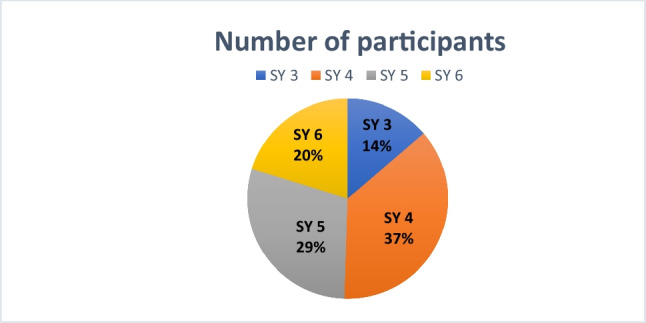
**Participant numbers in the study survey.** The figure illustrates the distribution of the 679 participants across the study years (SY 3 to SY 6)

Students from different study years (SY) participated in the survey. As shown in Fig. 2, 14% of respondents were in SY 3. The largest proportion of participants (37%) were in their fourth year. A further 29% were in their fifth year, and 20% were in their final clinical year (practical year, SY 6). The majority of respondents were therefore in advanced stages of their studies, which enhances the relevance of their evaluations regarding pharmacology education.

### Participating universities

The highest number of participants was recorded at MHH, with 167 students, followed by Leipzig University with 55 participants. There were 51 participants at the Georg-August-Universität Göttingen, 46 participants at the University of Cologne, 33 participants at the University of Duisburg-Essen, and 29 participants at the Westfälische Wilhelms-Universität Münster. Further participants came from Friedrich Schiller University Jena (27 participants), Humboldt University Berlin (25 participants), Otto von Guericke University Magdeburg, and the University of Lübeck, which each had 24 participants. The Eberhard-Karls-Universität Tübingen and the Ruhr-Universität Bochum each had 22 participants, followed by the Heinrich-Heine-Universität Düsseldorf and the Technische Universität Dresden with 18 participants each. The Johann Wolfgang Goethe University Frankfurt am Main and the University of Hamburg each had 14 participants. Lower numbers of participants were recorded at Justus Liebig University Giessen (12 participants), the Mannheim Medical Faculty of Heidelberg University, Ruprecht Karls University Heidelberg (10 participants each), and the University of Rostock (9 participants). There were even smaller numbers of participants at RWTH Aachen University (8 participants), Martin Luther University Halle-Wittenberg (7 participants), Christian-Albrechts University Kiel (6 participants), the Technical University of Munich (5 participants), and the University of Ulm (2 participants). Single responses were received from Friedrich Alexander University Erlangen-Nuremberg, the University of Freiburg, and Saarland University in Homburg (see Supplementary Table S1).

### Importance of pharmacology

**Fig. 3:**
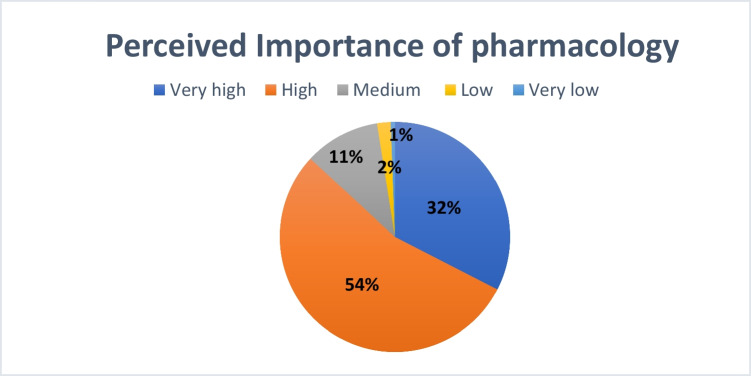
**Perceived importance of pharmacology.** The majority of respondents assessed the importance of pharmacology as “high” or “very high”

The survey asked students to evaluate the importance of pharmacology in medical education and its relevance to their future clinical careers across various universities (see Fig. 3). Among all respondents, 1% rated the importance of pharmacology as “very low,” 2% as “low,” and 11% as “moderate.” The majority (54%) rated it as “high,” while 32% selected “very high.”

Comparable findings were observed at MHH: 1% of participants rated pharmacology as “very low” in importance, 2% as “low,” 10% as “moderate,” 51% as “high,” and 36% as “very high” (see Supplementary Figure S2).

When excluding MHH, pharmacology remained predominantly rated as relevant (see Supplementary Figure S3): 56% of students from other institutions selected “high,” 31% “very high,” and 11% “moderate,” while only a small minority rated the subject as “low” (2%) or “very low” (0%).

At Leipzig University, 2% of students rated pharmacology as “very low,” 2% as “low,” 14% as “moderate,” 60% as “high,” and 22% as “very high” (see Supplementary Figure S4).

Overall, pharmacology was predominantly considered important at both institutions. However, the proportion of “very high” responses was higher at MHH (36%) compared to Leipzig (22%) and also slightly higher across all other universities combined (31%), which initially included Leipzig.

These findings are supported by the scientific literature, which highlights the central role of pharmacology in medical education. Maxwell and Walley (2003), for example, emphasized that solid pharmacological knowledge is essential for safe and effective patient care. They argued that understanding pharmacology is fundamental to sound clinical decisions. It is therefore seen as indispensable for future physicians.

However, such literature may not only reflect the objective importance of pharmacology. It may also shape curricular priorities and influence students’ perceptions. The strong emphasis on pharmacology at some institutions could be both a result of and a response to these frameworks. This suggests a reciprocal relationship between published recommendations and how students perceive the subject’s relevance.

### Evaluation of pharmacology teaching

Participants were also asked to assess the quality of pharmacology teaching at their respective universities. The aim was to identify institutional differences in perceived teaching quality and to draw conclusions about strengths and areas for improvement.

**Fig. 4:**
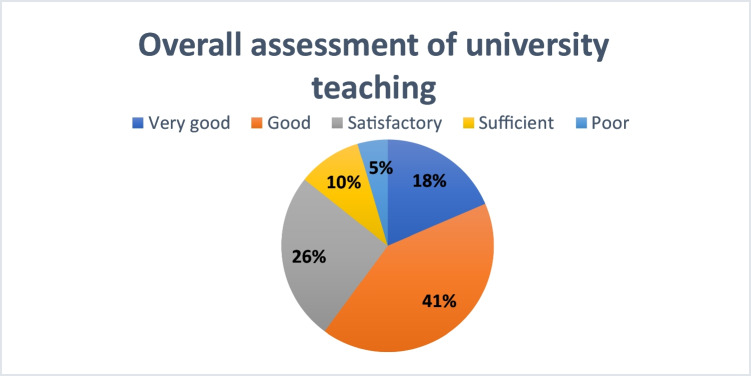
Overall assessment of university teaching in pharmacology

Across all respondents, 18% rated pharmacology teaching as “very good,” 41% as “good,” 26% as “satisfactory,” 10% as “sufficient,” and 5% as “poor” (see Fig. 4).

At MHH, 34% of students rated the teaching as “very good,” 39% as “good,” 18% as “satisfactory,” 5% as “sufficient,” and 4% as “poor” (see Supplementary Figure S5).

At universities excluding MHH, pharmacology teaching was mainly rated as “good” (42%) and “satisfactory” (28%), with fewer students selecting “very good” (14%), 11% as “sufficient,” and 5% as “poor” (see Supplementary Figure S6).

At Leipzig University, 11% rated the teaching as “very good,” 36% as “good,” 36% as “satisfactory,” 13% as “sufficient,” and 4% as “poor” (see Supplementary Figure S7).

The results indicate institutional differences in the perception of pharmacology teaching. MHH received favorable evaluations. In contrast, Leipzig was rated not only lower than MHH but also below the average of the other universities, suggesting comparatively lower levels of satisfaction.

### Preparation methods for the university pharmacology exam

**Fig. 5:**
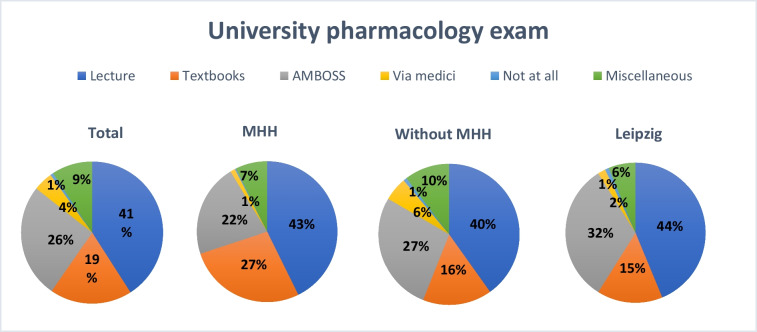
Distribution of the selected preparation methods for the university pharmacology exam overall, at MHH, at all universities excluding MHH, and at Leipzig University

The survey explored how students prepared for their university pharmacology exams (see Fig. 5). Participants were presented with multiple-choice options and had the opportunity to add open-text comments to provide a comprehensive view of their study strategies. Only 1% of respondents reported that they had not prepared for the exam. Via Medici was used by 4%, while 9% utilized other types of study materials. Textbooks were used by 19% of participants, whereas 26% relied on the AMBOSS platform. The majority of students (41%) reported preparing by attending lectures. These findings are consistent with the results of Wynter et al. (2019), who found that, despite the increasing use of digital learning tools, attending in-person lectures remains the most commonly used method for acquiring new material.

A comparable pattern was observed at MHH. None of the respondents reported not having prepared. Via Medici was used by 1%, other methods were reported by 7%, AMBOSS by 22%, textbooks by 27%, and 43% of students prepared for the examination by attending lectures.

Across all other participating universities (excluding MHH), a similar distribution of preparation strategies was observed. Lectures were the most frequently chosen method, reported by 40% of respondents. AMBOSS was used by 27%, textbooks by 16%, and 10% used other resources. Via Medici was selected by 6%, and only 1% reported not having prepared for the pharmacology exam.

At Leipzig University, lectures were likewise the most commonly used preparation method, reported by 44% of students. In addition, 32% used AMBOSS, 15% relied on textbooks, 2% used Via Medici, and 6% indicated using other resources. Only 1% of participants from Leipzig stated that they had not prepared.

Although three students did not complete the questionnaire in full, their answers were included in the analysis (see Supplementary Figure S8). The analysis of 136 open-text responses revealed that the most frequently mentioned resources were lecture notes or scripts (*n* = 27), past exam questions (*n* = 19), Meditricks (*n* = 16), and flashcards or Anki (*n* = 15). Other mentioned resources included textbooks (*n* = 10), lectures (*n* = 5), and problem-based learning (PBL) approaches (*n* = 4).

In summary, the results suggest that while digital platforms are widely used, traditional resources such as lecture notes and past exam questions, along with audiovisual tools like Meditricks, continue to play a central role in pharmacology exam preparation.

## Preparation methods for the second state examination

**Fig. 6:**
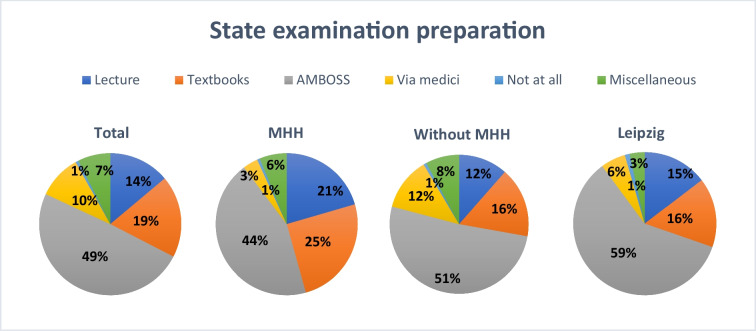
Preparation methods for the state examination overall, at MHH, at all universities excluding MHH, and at Leipzig University

Subsequently, students were asked which methods they use or intend to use to prepare for pharmacology in the second state examination (see Fig. 6). A multiple-choice format was employed to capture a broad spectrum of preparation strategies.

Of all respondents, 49% indicated that they used the AMBOSS platform to prepare for the exam. Textbooks were used by 19%, and 14% reported attending lectures. Via Medici was employed by 10% of students, while 7% mentioned alternative methods, including individual learning strategies. Only 1% stated that they did not prepare or did not intend to prepare for the pharmacology section of the exam.

A similar distribution was observed at MHH. Here, 44% of students reported using AMBOSS, 25% used textbooks, and 21% relied on lectures. Only 6% reported using other methods, 3% used Via Medici, and 1% indicated that they had not prepared for the second state examination.

A comparable distribution was observed at all other universities excluding MHH. Here, 51% of students reported using AMBOSS to prepare for the second state examination. Textbooks were used by 16%, 12% attended lectures, and 12% reported using Via Medici. Miscellaneous methods were indicated by 8% of respondents, and only 1% stated that they had not prepared.

At Leipzig University, 59% of students stated that they used AMBOSS, followed by 16% who used textbooks and 15% who prepared by attending lectures. Via Medici was selected by 6%, and 3% reported other methods. Similarly, 1% of respondents indicated no specific preparation.

In addition to these general approaches, some students (7%) mentioned alternative resources. Among this group, 47% used study notes, flashcards, or written summaries. Pharmacology textbooks and “Endspurt” scripts were used by 20%, while 17% utilized video-based learning resources. A further 9% used online materials, and 7% provided responses deemed irrelevant and excluded from analysis (see Supplementary Figure S9). Responses were considered irrelevant if they did not clearly relate to the research question.

A clear trend in the data indicates that AMBOSS is the dominant tool used for exam preparation. This applies both to the overall average and to the individual universities. While AMBOSS is the most frequently used resource at both sites, students at MHH are more likely to supplement it with textbooks and lectures. In contrast, students in Leipzig rely even more heavily on AMBOSS, with traditional teaching methods playing a lesser role. The results illustrate the central role of digital learning platforms in exam preparation, although there are location-specific differences in the use of supplementary methods.

This trend is further supported by recent user data: over 95% of medical students in Germany reportedly use the AMBOSS (2016) platform to prepare for their state examinations. Additionally, 24 out of 37 medical faculties provide institutional access to AMBOSS, reflecting its widespread use and reinforcing the patterns observed in this study.

In the context of preparing for the second state examination, AMBOSS is by far the most frequently used learning tool, mentioned in nearly 50% of responses. The ability to directly link content knowledge with the application of past state examination questions appears particularly attractive. Platforms such as AMBOSS offer structured study plans for this purpose. For example, the 100-day learning plan combines daily study units with exam questions and is specifically tailored to the requirements of the state examination. This approach aligns with the findings of Wynter et al. (2019), who demonstrated that question banks, whether online or downloadable, are among the most commonly used revision tools. Students particularly value the opportunity to test their knowledge using previous exam questions and to create a direct connection between study content and the exam format.

### Intensity of involvement with pharmacology

**Fig. 7:**
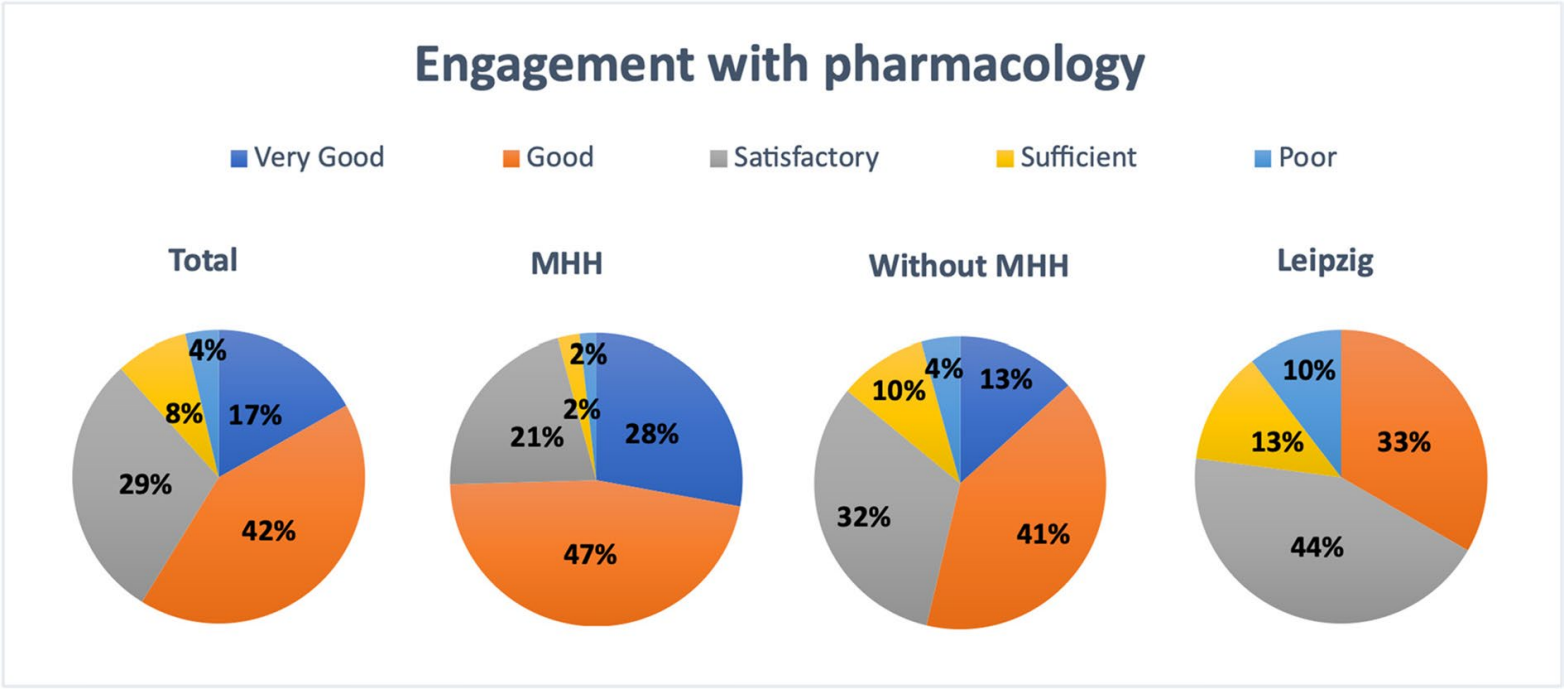
Analysis of engagement with pharmacology based on survey results, presented overall, at MHH, at all universities excluding MHH, and at Leipzig University

The question of how intensively students had engaged with pharmacology yielded the following results across all respondents: 17% stated that their engagement was rated as “very good,” 42% as “good,” 29% as “satisfactory,” 8% as “sufficient,” and 4% as “poor” (see Fig. 7).

At MHH, 28% of participants reported “very good” engagement, 47% “good,” 21% “satisfactory,” and only 2% rated it as “sufficient” or “poor.”

Among participants from universities excluding MHH, 13% reported a “very good” engagement with pharmacology, while 41% described it as “good.” Thirty-two percent rated their engagement as “satisfactory,” 10% as “sufficient,” and 4% as “poor.”

In contrast, a markedly different pattern emerged at Leipzig University. None of the participants rated their engagement with pharmacology “very good.” A total of 33% indicated “good” engagement, 44% rated it as “satisfactory,” and 13% “sufficient.” Approximately one in ten respondents stated that their engagement with the subject as “poor.”

### Use of pharmacology textbooks

A central component of the survey was to ascertain which textbooks students used to prepare for the second state examination in pharmacology. The results show that the majority of respondents did not use textbooks, while others reported using specific titles. The data indicate significant variation in textbook selection between MHH and the other participating universities.

**Fig. 8:**
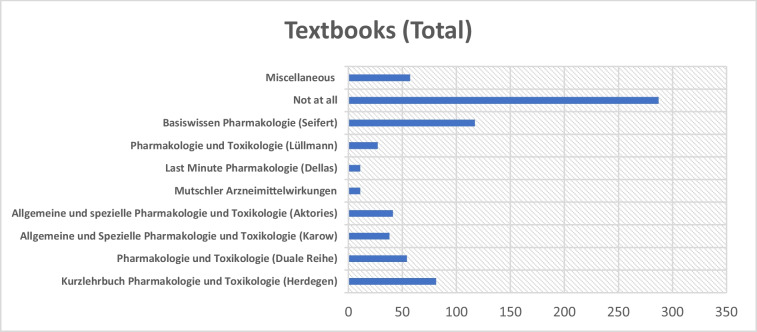
Distribution of textbooks used in the overall average in the survey. The *x*-axis indicates the number of students who reported using each textbook

In total, 287 participants indicated that they had not used a textbook for exam preparation (see Fig. 8). Among those who did, 117 referred to “Basiswissen Pharmakologie” by Seifert. The “Kurzlehrbuch Pharmakologie und Toxikologie” by Herdegen was used by 81 students, while 54 relied on the Duale Reihe “Pharmakologie und Toxikologie.” Around 38 participants reported using Karow’s and 41 Aktories’ “Allgemeine und Spezielle Pharmakologie und Toxikologie.” The textbook by Lüllmann was cited by 27 students. A smaller number used “Mutschler Arzneimittelwirkungen” (11) or “Last Minute Pharmakologie” by Dellas (11). Additionally, 57 respondents mentioned other materials, indicating the use of a diverse range of supplementary resources (see below).

**Fig. 9:**
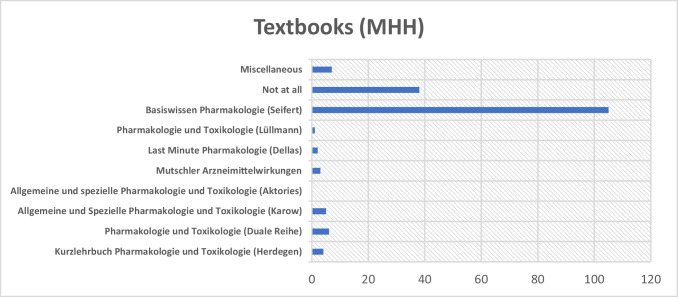
Distribution of textbooks used at MHH. The *x*-axis indicates the number of students who reported using each textbook

MHH showed a distinct trend in students’ textbook preferences: 105 participants stated that they had prepared themselves using Seifert’s textbook. Thirty-eight participants did not use any textbook at all. Other textbooks were only used by a very small number of students, whereby it is striking that none of the respondents used the work “Allgemeine und Spezielle Pharmakologie und Toxikologie” by Aktories for exam preparation (see Fig. 9).

This distribution might be explained by Professor Seifert’s close academic involvement in pharmacology teaching at MHH, which may have influenced students’ textbook choices. This institutional proximity could have led to a certain bias in the responses.

**Fig. 10:**
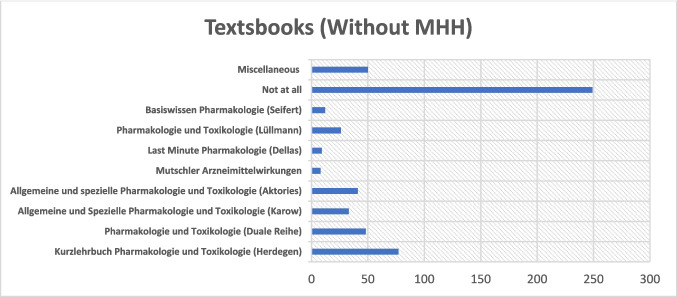
Distribution of textbooks used (excluding MHH results). The *x*-axis indicates the number of students who reported using each textbook

Among participants from universities excluding MHH, 249 stated that they had not used any textbook for exam preparation (see Fig. 10). The “Kurzlehrbuch Pharmakologie und Toxikologie” by Herdegen was used by 77 students, while 48 relied on the Duale Reihe “Pharmakologie und Toxikologie.” The textbook by Aktories was used by 41 students, and that by Karow by 33 students. The textbook by Lüllmann was cited by 26 students. “Basiswissen Pharmakologie” by Seifert was cited by 12 students. A smaller number used “Mutschler Arzneimittelwirkungen” (*n* = 8) or “Last Minute Pharmakologie” by Dellas (*n* = 9). Additionally, 50 respondents mentioned other materials, indicating the use of a diverse range of supplementary resources.

**Fig. 11:**
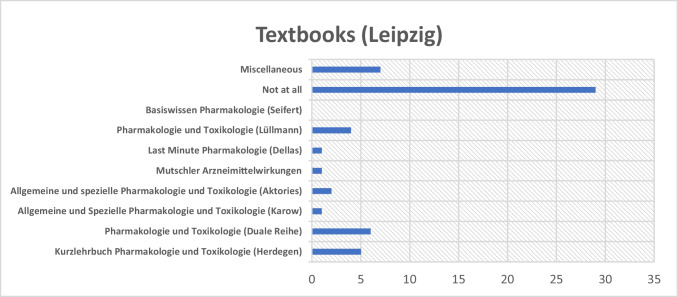
Distribution of textbooks used at Leipzig University. The *x*-axis indicates the number of students who reported using each textbook

A different distribution was observed at Leipzig University (see Fig. 11). Most respondents there also reported preparing without any textbook. Among those who did use a textbook, there was no clear preference for a specific title. Notably, none of the students selected Seifert’s “Basiswissen Pharmakologie.” Instead, respondents more frequently indicated the use of alternative materials, such as the Duale Reihe “Pharmakologie und Toxikologie” or Herdegen’s “Kurzlehrbuch Pharmakologie und Toxikologie.”

Observed patterns may reflect institutional differences in students’ textbook use. At MHH, textbook-based preparation appears more consistent, with a strong preference for Seifert’s textbook. In contrast, students at other universities reported using a wider range of resources and more often prepared without relying on a specific textbook. This may indicate either a greater openness to alternative learning materials or a more decentralized approach to textbook guidance.

Participants were also provided with an open-text option to list additional resources (see Supplementary Figure S10). The qualitative analysis of these responses revealed that 14 entries were excluded due to irrelevance. Among the remaining responses, 11 students cited “Last Minute Pharmakologie” by Dellas, 7 mentioned the textbook by Freissmuth, and 5 referred to the Endspurt scripts. Three respondents used Meditricks materials. Furthermore, two students each indicated the use of Arzneimittelpocket or English-language literature, while one student mentioned “Klinische Pharmakologie*”* by Wehling and another cited Ellegast’s textbook.

### Use of English-language literature

The survey investigated whether students referred to English-language literature during their exam preparation. In total, 2% of respondents indicated that they had used such resources (see Supplementary Figure S11). The materials most frequently mentioned included online learning platforms, PubMed articles, peer-reviewed publications, and educational video content (see Supplementary Figure S12). Interestingly, three respondents specifically referred to the English edition of Seifert’s textbook. This may be linked to his institutional affiliation with MHH and could have introduced a degree of bias in the responses. Furthermore, one participant each reported using Netter’s Illustrated Pharmacology, Goodman and Gilman’s, Katzung’s Pharmacology, and First Aid 2020. One entry was excluded from the analysis as it did not pertain to the topic.

### Requirements for modern pharmacology textbooks

**Fig. 12:**
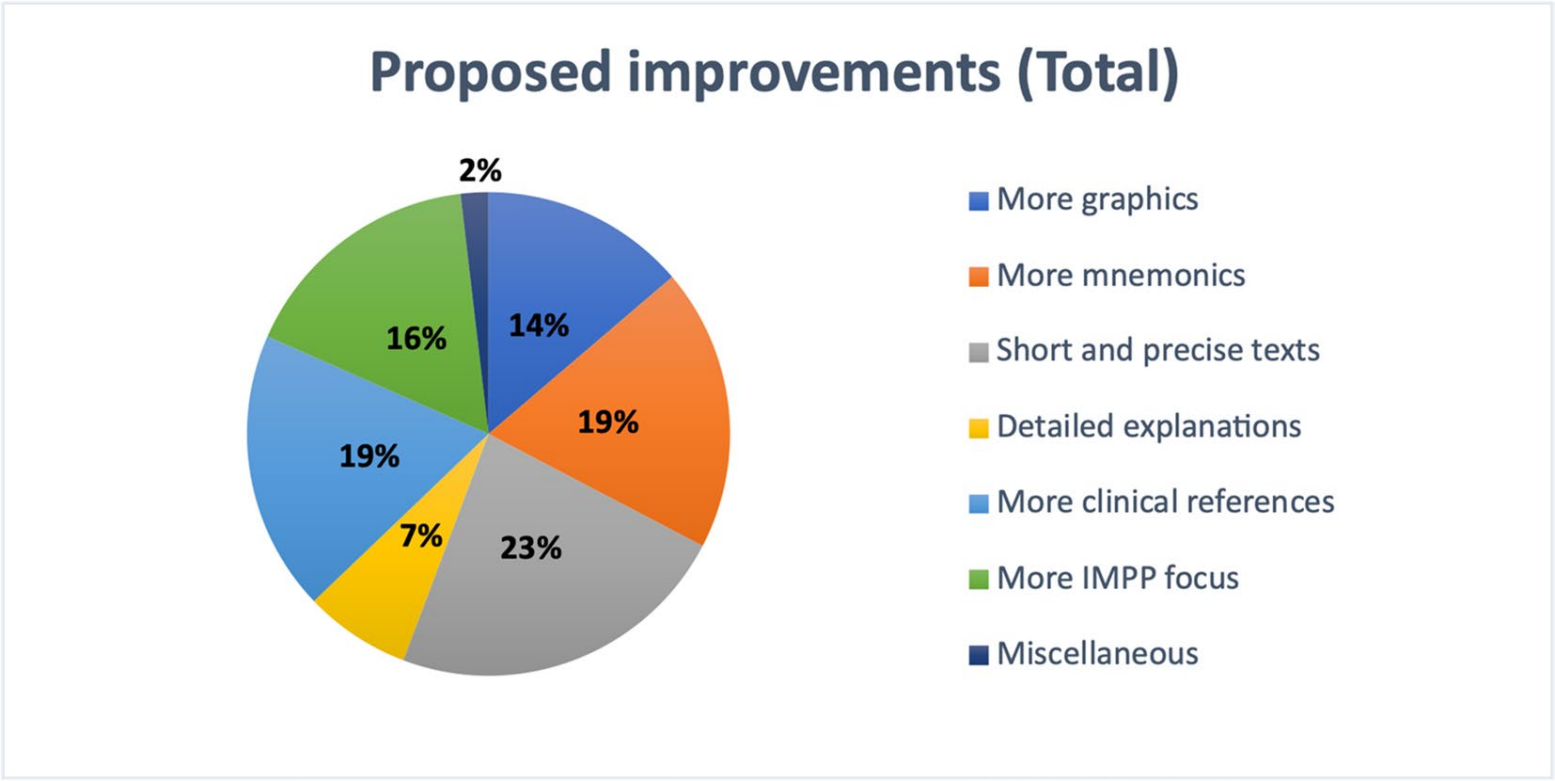
Overall student-reported suggestions for improving textbooks

The survey went on to investigate which factors would motivate students to use pharmacology textbooks for exam preparation (see Fig. 12). Respondents could indicate up to three factors that would be most important to them.

The most important feature (23%) for the respondents was short and clearly structured texts. In each case, 19% of respondents felt that mnemonics and a stronger clinical reference were important. A greater emphasis on Institute for Medical and Pharmaceutical Proficiency Assessment (IMPP)-style questions was identified as important by 16%, while 14% valued enhanced visual presentation through illustrations. More detailed explanations were preferred by 7%, and 2% expressed other preferences.

The widespread preference for increased clinical relevance highlights the importance of interactive and applied learning strategies in pharmacological education. Fasinu and Wilborn (2024) demonstrated that interactive, problem-based, and case-based learning formats can significantly enhance conceptual understanding in pharmacology.

The results of Michel et al. (2002) further support the survey’s findings regarding preferences for practical learning approaches. In their study, a pharmacology course for third-year medical students was evaluated by comparing PBL with traditional lecture-based learning (LBL). Students in the PBL group worked in small teams on clinical case studies and also attended supplementary lectures. The LBL group, on the other hand, learned through lectures only. Students in the PBL group performed better on the final pharmacology exam and had a lower failure rate than those in the LBL group. They also reported a higher interest in pharmacology and indicated a better conceptual understanding. Overall, the study shows that PBL can improve both motivation and learning outcomes, while still ensuring the acquisition of essential knowledge.

**Fig. 13:**
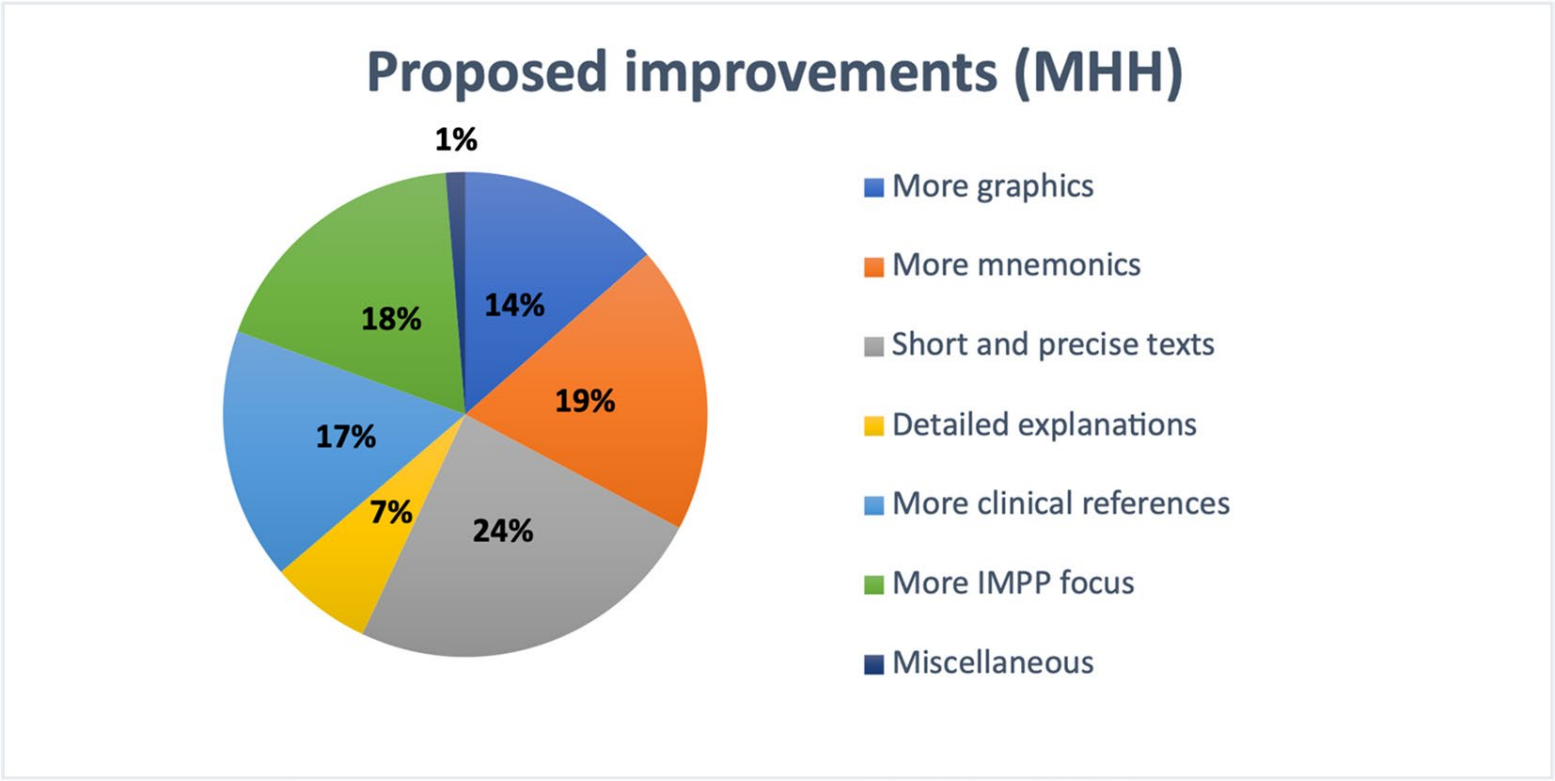
Student-reported textbook improvement preferences at MHH

A similar pattern was seen at MHH (see Fig. 13). Here, 24% of students preferred short and clear texts. Another 19% valued the use of mnemonics, while 18% found a stronger focus on IMPP-related content important. Clinical relevance was highlighted by 17% of participants, 14% wanted more visual content such as graphics, and 7% asked for more detailed explanations. Only 1% of respondents mentioned other individual preferences.

**Fig. 14:**
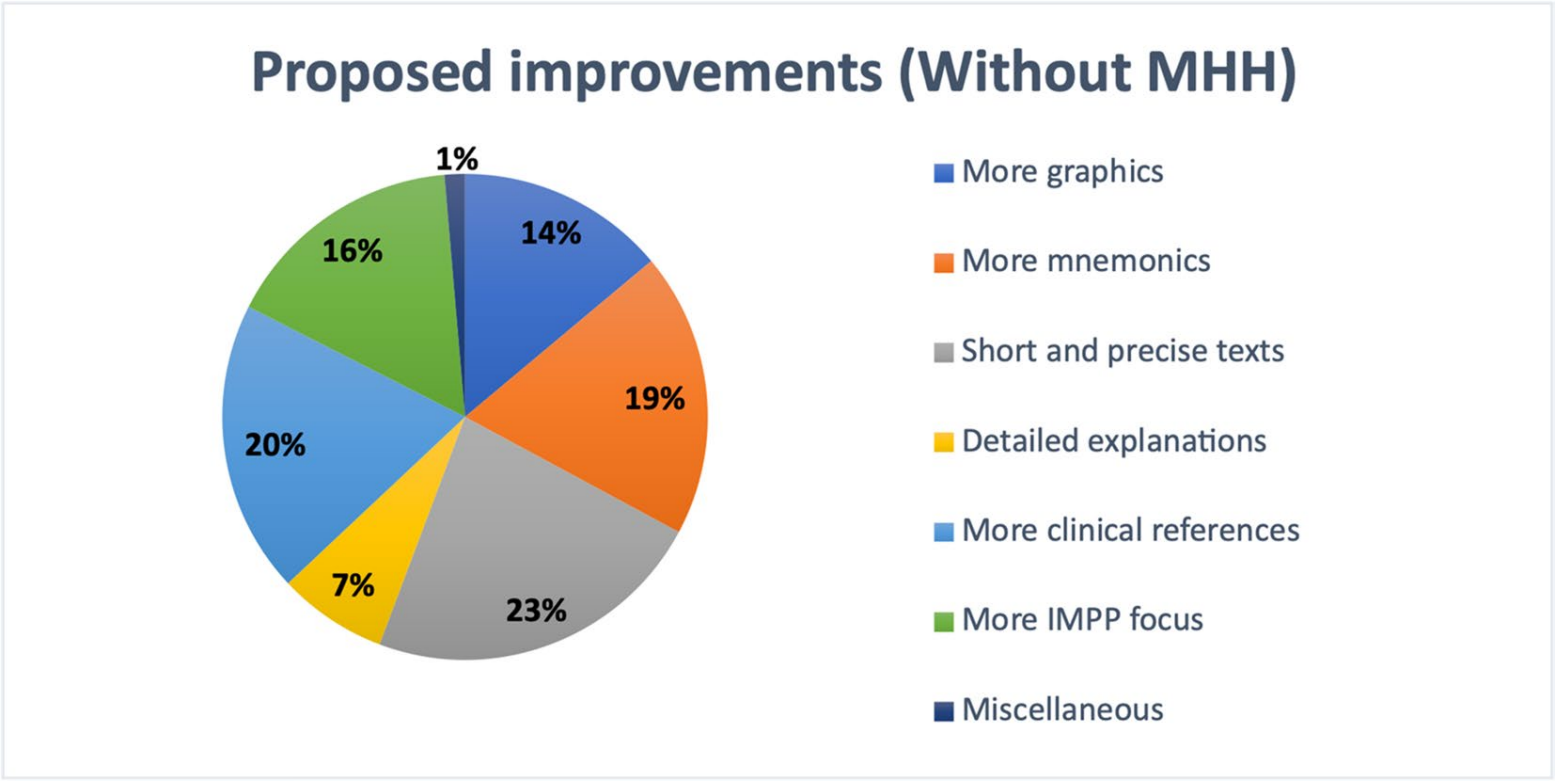
Student-reported textbook improvement preferences at universities excluding MHH

At the other participating universities excluding MHH, 23% of students suggested the use of short and precise texts as the most important improvement (see Fig. 14). Clinical relevance was emphasized by 20%, while 19% valued the use of mnemonics. A stronger focus on IMPP-related content was requested by 16% of respondents, 14% wished for more graphics, and 7% asked for more detailed explanations. Only 1% mentioned other individual suggestions.

**Fig. 15:**
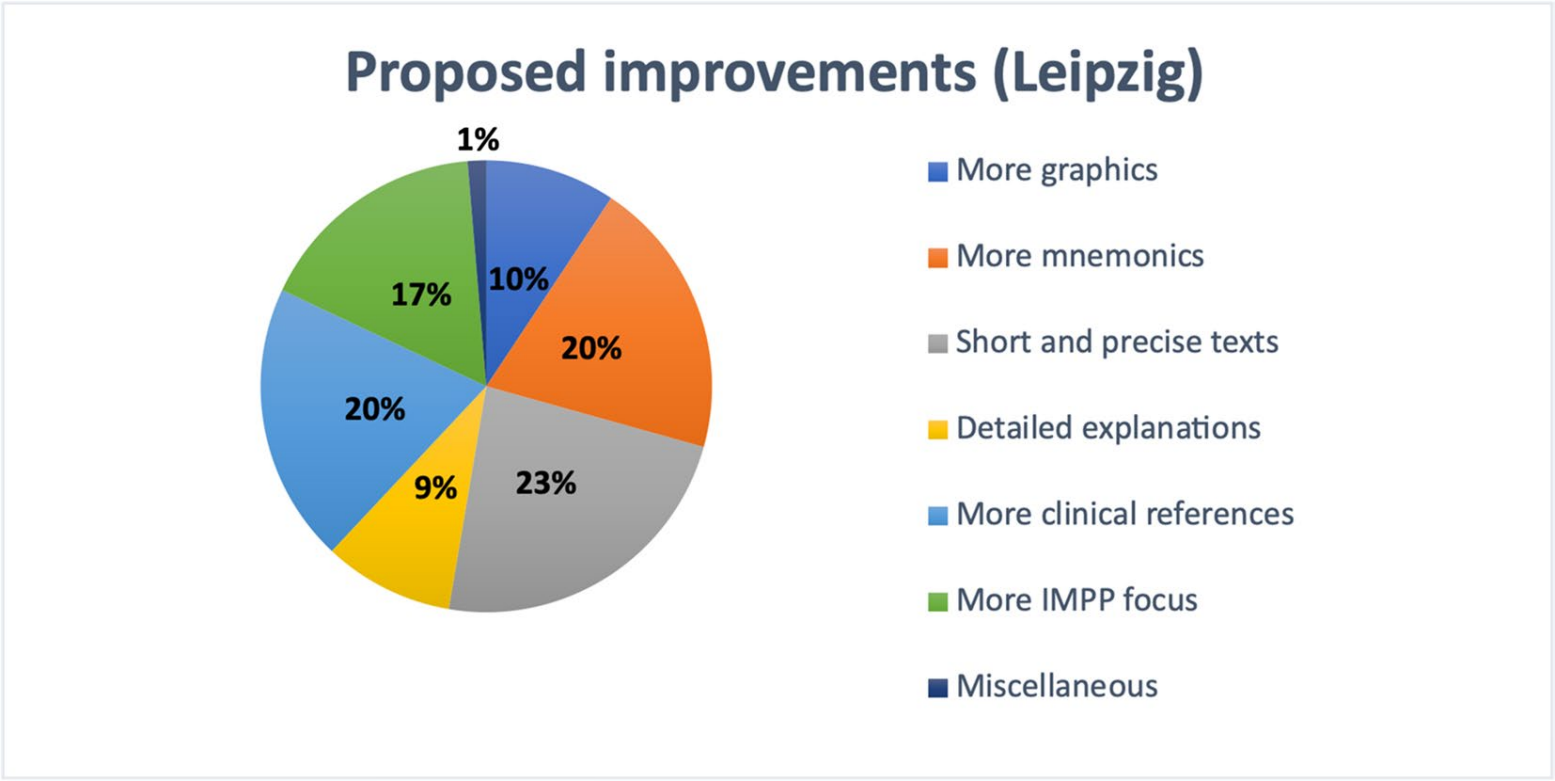
Student-reported textbook improvement preferences at Leipzig University

At Leipzig University, responses followed a comparable trend (see Fig. 15). A total of 23% of students highlighted the value of short and clear texts. Twenty percent favored the use of mnemonics, and another 20% preferred more clinically relevant content. A stronger focus on IMPP topics was important to 17%, while 10% requested more graphics and 9% wished for more detailed explanations. A small number of participants mentioned other individual preferences.

Among the open-text responses categorized as “Other,” three participants pointed out the importance of up-to-date content and the limited time available during medical studies. Two students each expressed a preference for well-balanced text length, visual learning aids, clear tables, deeper explanations, and the option to access textbooks in PDF or online formats. Individual responses included wishes for textbooks such as First Aid*,* note-taking place, affordable pricing, and better coordination between lectures and textbook material. Further suggestions included audiobooks, compact summaries, more clinically relevant material, and the integration of clinical case examples (see Supplementary Figure S12).

Fasinu and Wilborn (2024) highlighted the effectiveness of interactive learning tools such as online quizzes, instructional videos, and educational games like Kahoot or Jeopardy in enhancing student motivation and participation. Pharmacology textbooks could integrate these tools by embedding QR codes or hyperlinks that lead to supplementary digital resources. Audience Response Systems (ARS) may also be incorporated via dedicated platforms. These tools were found to support a more visual, dynamic, and engaging learning experience, which many students consider important in their exam preparation.

### Assessment of preparation for professional practice

The survey also explored the extent to which students felt that their pharmacology education had prepared them for professional life. Responses were collected using a single-choice format.

**Fig. 16:**
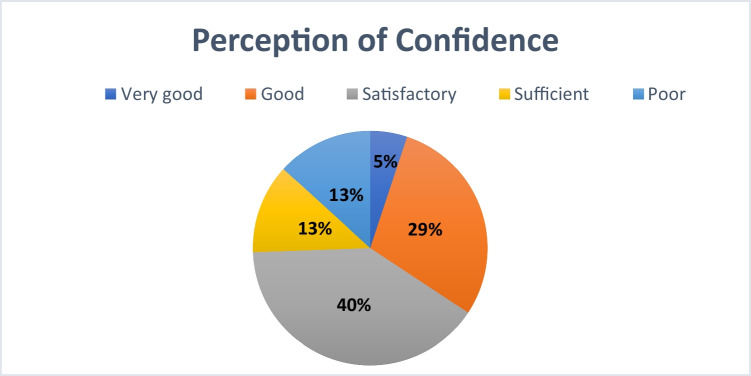
Perception of confidence in the field of pharmacology based on overall survey results

In total, 5% of participants rated their perception of confidence as “very good.” Most students selected either “good” (29%) or “satisfactory” (40%). An additional 13% rated it as “sufficient,” and another 13% as “poor” (see Fig. 16).

The findings of the survey indicate that most students feel only moderately prepared for their future professional responsibilities. Notably, only a small proportion of respondents considered themselves very well prepared. In this context, the observations made by Hafner et al. (2022) regarding the use of digital learning platforms during the COVID-19 pandemic are relevant. The authors note that although these platforms enabled efficient distribution of learning materials, heavy reliance on them resulted in a narrower learning focus, often limited to prestructured content. They further emphasize that a well-rounded medical education, especially in the area of clinical pharmacology, requires additional competencies, such as the ability to independently identify and evaluate evidence-based information.

The article specifically references MHH, where a data literacy elective was introduced in 2020. This course is intended to foster the development of relevant skills and may help explain the comparatively higher satisfaction reported by MHH students. At MHH, 9% of respondents rated their preparation as “very good,” 42% as “good,” 35% as “satisfactory,” 6% as “sufficient,” and 8% as “poor” (see Supplementary Figure S14).

Among participants from all other universities excluding MHH, 4% felt “very good” prepared for practical pharmacology application (see Supplementary Figure S15). Twenty-five percent rated their preparation as “good,” 42% as “satisfactory,” 14% as “sufficient,” and 15% as “poor.”

By contrast, students at Leipzig University reported feeling less well prepared (see Supplementary Figure S16). None of the participants described their preparation as “very good.” While 24% felt “good,” 44% rated it as “satisfactory,” 15% as “sufficient,” and 17% as “poor.”

These observations align with the findings of Brinkman et al. (2017), who reported that many medical students lack essential competencies in clinical pharmacology and pharmacotherapy, contributing to a general sense of unpreparedness for clinical duties.

To further address whether students who reported textbook use represent a distinct group, subgroup analyses were conducted based on textbook usage (see Supplement Figures S17–S24). A total of 142 students reported having used textbooks for both university exam preparation and the second national licensing examination. Additionally, 84 students planned to use textbooks exclusively for the licensing exam, while 132 students reported textbook use only in preparation for university exams. Furthermore, 321 students stated that they did not use textbooks for either exam.

Regarding perceived preparedness in pharmacology, the most favorable response pattern was observed among students who used textbooks for both exams (see Supplementary Figure S17): 38% reported feeling “good,” 36% “satisfactory,” and 9% “sufficient,” while 11% rated their confidence as “very good” and only 6% as “poor.” A similar distribution was found among students who used textbooks exclusively for university exams (see Supplementary Figure S18): 4% reported “very good,” 38% “good,” 36% “satisfactory,” and 11% each “sufficient” and “poor.” In contrast, students who used textbooks only for the state exam (see Supplementary Figure S19) showed a lower overall confidence level: 4% reported “very good,” 24% “good,” 39% “satisfactory,” 15% “sufficient,” and 18% “poor.” Students who did not use textbooks at all (see Supplementary Figure S20) also showed higher proportions of “poor” (14%) and “sufficient” (14%), with 44% selecting “satisfactory,” 23% “good,” and only 5% “very good.”

With respect to the perceived importance of pharmacology, students who used textbooks for both exams (see Supplementary Figure S21) showed strong appreciation: 38% rated the subject as “very high,” 49% as “high,” 9% “moderate,” 3% “low,” and 1% as “very low.” Among those who used textbooks only for university exams (see Supplementary Figure S22), 38% selected “very high,” 54% “high,” 7% “moderate,” 1% “low,” and no one selected “very low.” Students who used textbooks only for the licensing exam (see Supplementary Figure S23) rated it slightly lower, with 33% “very high,” 54% “high,” 12% “moderate,” 1% “low,” and no “very low.” In comparison, non-users (see Supplementary Figure S24) showed the lowest proportion of “very high” ratings (28%), while the majority (57%) selected “high,” followed by 12% “moderate,” 2% “low,” and 1% “very low.”

Students who used textbooks during both examination phases reported greater confidence in their pharmacological competence and perceived the subject as more relevant. In contrast, those who did not use textbooks felt less prepared and rated pharmacology as less important. While this association does not imply causality, it may reflect differences in study strategies, learning preferences, or perceived curricular expectations.

## Conclusions and integration with relevant literature

This study on the use of pharmacology textbooks in exam preparation shows that such resources still play a relevant role. However, they are increasingly being replaced or complemented by digital learning tools. While some students consider textbooks useful for gaining a deeper understanding of pharmacological content, others view them as less efficient, particularly when compared to interactive digital formats that offer greater flexibility and more up-to-date information. These findings suggest that traditional textbooks no longer fully meet the current learning preferences of many students.

The relevance of digital and mixed learning formats was also highlighted by Servos et al. (2023). They studied a PBL approach that combined an initial online meeting via chat with a later in-person session. Students in this group achieved exam results similar to those of a traditional face-to-face group. Although the online chat was seen as less interactive than in-person meetings, this did not negatively affect learning outcomes or evaluations. These results suggest that digital teaching methods can be effective without reducing the quality of education.

The benefits of self-directed learning (SDL) in medical education have been well documented. In a widely cited study by Parker and Mazmanian (1987), four practicing physicians in community hospitals developed individualized learning contracts in collaboration with clinical experts. These contracts included specific goals in internal medicine, and participants independently selected their learning resources, such as seminars and self-study materials. The intervention was associated with measurable knowledge gains and reported improvements in clinical practice and patient care.

A systematic review by Murad et al. (2010) further supports the effectiveness of SDL in health professions education. The authors found that SDL can be equally or more effective than traditional instructional methods, particularly when learners are actively engaged in identifying their own educational needs and selecting appropriate resources. The review concluded that involving learners in the planning and implementation of their learning enhances motivation and supports improved learning outcomes.

The survey further showed that students prefer content to be concise and clearly structured. Long or overly detailed texts were often viewed as discouraging. Focusing on key concepts and using memorable phrases may help improve engagement and comprehension. Students also expressed a desire for closer integration of theory and practice. Case-based learning and clinically relevant content may help them apply knowledge more effectively to real-life scenarios, potentially improving professional readiness and addressing existing gaps in clinical pharmacology and pharmacotherapy.

Moreover, the survey highlighted a wish for a stronger focus on exam-relevant content, particularly questions from the IMPP. To support this, teaching materials should be more clearly adapted to the structure and demands of the examinations. There was also a noticeable preference for increased visual content, such as diagrams and illustrations, which may help simplify complex topics and improve comprehension.

Students reported clear preferences regarding the structure and layout of pharmacology textbooks. While these preferences provide valuable insights for textbook development, they should not serve as the sole basis for instructional decisions. Educational materials should be designed based on established principles that promote effective learning.

Although recent research has primarily focused on teaching methods, some findings are also relevant for textbook design. For instance, a study by Xiao et al. (2023) evaluated various teaching strategies in pharmacology education and found that approaches fostering active engagement and cognitive processing were associated with improved learning outcomes and higher student satisfaction. While the study did not assess textbooks directly, it supports the broader principle that learning materials should be structured to facilitate deep understanding and active learning.

Survey results indicated that textbooks were used at similar rates for both university examinations and the second national licensing examination. Frequently mentioned titles included “Basiswissen Pharmakologie” and “Kurzlehrbuch Pharmakologie und Toxikologie.” However, the role of textbooks appears to shift throughout the course of medical training. In the context of university examinations, textbooks are likely used for detailed and structured study of individual subjects.

By contrast, during preparation for the licensing exam, they may serve primarily as supplementary references alongside online learning platforms. At this stage, resources such as AMBOSS play a more central role, offering compact content and question banks aligned with exam requirements. In addition to its learning cards and IMPP-style questions, AMBOSS provides a range of supplementary features that can support individual exam preparation. Some of these resources are available through campus licenses or can be unlocked via additional modules, including Meditricks, Smart Histology and Pathology, and the Human Body Explorer (AMBOSS (2025a)). AMBOSS also offers downloadable Anki decks covering both preclinical and clinical content (AMBOSS (2025b)). In addition, the Auditorium format presents selected medical topics as short audio recordings (AMBOSS (2025i, j, k, l, a, b, c, d, e, f, g, h)).

For users interested in staying up to date with current developments, AMBOSS offers two optional email services: the Studien-Telegramm (AMBOSS (2025c)), which summarizes recent publications in internal medicine, and the Leitlinien-Telegramm (AMBOSS (2025d)), which highlights updates to clinical practice guidelines. Furthermore, certain content is available in audio format (AMBOSS (2025e)). AMBOSS also produces the podcast AMBOSS Wissen, which presents clinical and academic topics in spoken form (AMBOSS (2025f)).

For pharmacological reference, AMBOSS integrates the full drug database provided by the ifap institute. This database includes information on active substances, potential interactions, dosing recommendations, and legal classifications (AMBOSS (2025g)). Additionally, online lectures are offered on a regular basis to complement existing study resources. Depending on the event, access may require an AMBOSS account. Whether participation is free or subject to fees depends on the specific format (AMBOSS (2025h)).

The AMBOSS GPT tool allows users to access platform content through a text-based interface using artificial intelligence (AMBOSS (2025i)). Certified continuing education formats are also available and meet the legal requirements for medical training in Germany (AMBOSS (2025j)). The browser extension AMBOSSify enables users to link medical terms encountered while browsing the internet directly to the relevant AMBOSS chapters (AMBOSS (2025k)).

Only a small number of students reported using English-language textbooks, indicating that German-language materials remain dominant and may benefit from further development.

From a didactic perspective, these patterns reflect the concept of constructive alignment. Students adapt their learning strategies to the expected cognitive demands, structure, and format of each assessment. University examinations differ between institutions and often focus on subject-specific knowledge. In comparison, the national licensing examination follows a standardized format and places greater emphasis on clinical reasoning and factual recall. Accordingly, students’ resource selection evolves during their studies.

In addition to AMBOSS, other platforms are available. Via medici offers structured study plans, practice questions modeled after IMPP exams, and access to textbooks from the Thieme publishing group. The MEDI-LEARN Repetitorium provides an external exam preparation program that supports students with thematically organized sessions, recorded webinars, and analyses of previous exams.

One limitation of this analysis is that it remains unclear why some students prepared using textbooks while others did not. Moreover, the survey did not assess whether textbook use was associated with improved or more structured pharmacological knowledge. This limits the interpretation of textbook-related findings. Future research could address this by including objective knowledge assessments or correlating resource usage with exam performance.

Another important aspect of the study concerned how well students felt prepared for their future professional roles through their pharmacology education. Many participants expressed uncertainty about their practical preparation. These findings are supported by Johannsen et al. (2019), who surveyed medical students across different semesters as well as alumni. The results showed that students consistently rated their applied pharmacological knowledge as insufficient throughout their studies. Although PBL was implemented, students’ confidence in applying knowledge to clinical practice remained low. In contrast, alumni rated their skills more positively, suggesting that real-world experience plays a crucial role in developing professional competence.

Another study by Kirsch and Matthes (2021) further highlights the value of practice-based teaching approaches. It showed that medical students who actively engaged in a simulation-based prescription exercise achieved significantly higher test scores than those who only observed or participated without simulation. Students who assumed the role of the physician gained the most from this hands-on experience. These results underscore the importance of practical teaching methods for building clinical skills.

The study by Sandholzer et al. (2014) reflected this development: 64.2% of medical students use smartphones, and 32.4% already rely on medical apps, mainly for drug information, diagnostic guidelines, and treatment instructions. Particularly high demand exists for resources related to medication, differential diagnosis, and visual content such as medical images and videos. On average, students reported a willingness to pay €14.35, indicating the perceived benefit of these digital tools.

In summary, this study provides useful insights for the future design of educational resources in pharmacology. The findings support a hybrid model that combines traditional and digital formats as a promising approach to meet the changing needs of medical students.

## Limitations

This study has several methodological limitations. Since participation was voluntary, there is a risk of self-selection bias, meaning that students with particularly strong opinions on teaching quality or specific learning methods may be overrepresented. The study was also limited to medical students in Germany, which restricts the generalizability of the findings to other countries with different curricula. Another limitation is the lack of a long-term perspective. The survey was conducted at a single point in time (October–November 2022), which means changes over the course of studies, such as shifts in textbook usage, could not be observed. The evaluation of teaching quality was based solely on participants’ subjective assessments, as no objective performance data were collected. Curricular differences between universities might have also influenced the results.

Moreover, a significant proportion of participants were enrolled at Hannover Medical School, where one of the authors, Professor Roland Seifert, teaches pharmacology. This connection may have influenced the high usage rate of the textbook “Basiswissen Pharmakologie,” which he authored. The authors recognize this potential conflict of interest and the resulting bias in textbook selection. All responses were submitted anonymously and voluntarily, and no guidance or incentives regarding textbook choices were provided. Nevertheless, the institutional overrepresentation and the possible influence of the author’s affiliation should be taken into account when interpreting the results. Additionally, results from Hannover Medical School were compared to those from all other universities to enable a more precise and differentiated analysis. Furthermore, it was not possible to determine which edition of each textbook was used by the participants. Therefore, an overview of the most recent editions available at the time of the survey (October 2022) has been compiled and presented in Supplementary Figure S25.

## Conclusions and future studies

The findings of this study indicate a noticeable shift in how medical students approach learning in pharmacology. Digital learning platforms, especially AMBOSS, have become the main study resource, gradually replacing traditional textbooks as the primary source of information. Key reasons for this shift include greater flexibility in time management, regularly updated content, and a stronger emphasis on topics relevant to examinations.

Hafner et al. (2022) observed a comparable development. At the beginning of the pandemic, many students still used both printed and digital learning materials. However, as online teaching became more established and access to libraries and copy shops was restricted, students increasingly relied on the resources provided through the learning management system, even though e-books and publicly available treatment guidelines remained accessible.

Despite the shift toward digital platforms, traditional textbooks continue to play an important role, particularly for developing a deeper conceptual understanding. However, these resources should be more closely aligned with students’ evolving learning preferences. A clear and concise structure, combined with visual elements and practical content, could enhance the long-term usefulness of conventional textbooks.

Despite this trend, traditional textbooks continue to play an important role, particularly for fostering conceptual understanding. Unlike online platforms that concentrate on exam preparation, textbooks may fulfill a broader educational function. They support long-term knowledge acquisition and conceptual understanding beyond licensing requirements. To maintain their relevance, textbooks could include clearly marked IMPP-related sections, concise summaries of essential points, and well-structured overviews. This strategy may help reconcile deep learning goals with exam-oriented efficiency. Rather than replacing digital resources, modernized textbooks could provide meaningful support across all stages of medical education.

Although 86% of students rated pharmacology as highly or very highly relevant to their future profession, only 59% reported high levels of engagement with the subject. This discrepancy may indicate that strong perceived importance does not necessarily translate into active study behavior. Several factors may contribute to this divergence, including disappointment with lecture quality, limited curricular integration, the perception that pharmacology is easy to pass, or the declining proportion of “pure” pharmacology questions in the state examination. Additionally, time constraints and the growing dominance of compact digital resources such as AMBOSS may lead students to deprioritize in-depth textbook learning. Further research is needed to identify which subgroups disengage and why, and whether curriculum design, assessment formats, or instructional quality contribute to this pattern.

Future strategies should focus on integrating hybrid learning models that combine the structured presentation of content in traditional textbooks with the benefits of digital learning tools. A modern design with clear visuals and regular updates based on current exam standards and clinical guidelines could help maintain the long-term usefulness of printed materials while supporting digital formats. Incorporating interactive components such as clinical case examples, QR codes, or online tasks may further enhance the educational quality of these resources.

In the long term, such a hybrid model seems to be a suitable way to provide students with solid knowledge in pharmacology and practical skills for real-life situations, helping them to be well prepared for the challenges of professional practice.

## Supplementary Information

Below is the link to the electronic supplementary material.

Supplementary file1 (DOCX 73.8 KB)

## Data Availability

All source data for this study are available upon reasonable request from the authors.

## Abbreviations

ARS: Audience Response Systems
IMPP: Institute for Medical and Pharmaceutical Proficiency Assessment
LBL: Lecture-based learning
MHH: Hannover Medical School
PBL: Problem-based learning
SDL: Self-directed learning
SY: Study year
