# Supplementary material for: What role do traditional pharmacology textbooks play in medical students’ education and exam preparation?
Source: Naunyn Schmiedebergs Arch Pharmacol. 2025 Sep 1;399(2):2547–64. doi: 10.1007/s00210-025-04459-3 (PMC12901173; doi:10.1007/s00210-025-04459-3)
Supplement: Supplementary file 1 — Supplementary file1 (DOCX 73.8 KB) [file 210_2025_4459_MOESM1_ESM.docx]

**What role do traditional pharmacology textbooks play in medical students' education and exam preparation?**

**Delany Manotheepan and Roland Seifert**

**Supplemental material**

**S1: Number of Participants per University**
The chart displays the number of survey participants from various universities. The data reveal an unequal distribution of participant numbers across the universities.

| **University** | **Number of Participants** |
| --- | --- |
| Friedrich Schiller University Jena | 27 |
| Friedrich-Alexander University Erlangen-Nürnberg | 1 |
| Goethe University Frankfurt | 14 |
| Hannover Medical School | 167 |
| Heidelberg University | 10 |
| Heinrich Heine University Düsseldorf | 18 |
| Humboldt University of Berlin | 25 |
| Johannes Gutenberg University Mainz | 15 |
| Justus Liebig University Giessen | 12 |
| Kiel University | 6 |
| Leipzig University | 55 |
| Martin Luther University Halle-Wittenberg | 7 |
| Medical Faculty Mannheim, Heidelberg University | 10 |
| Otto von Guericke University Magdeburg | 24 |
| RWTH Aachen University | 8 |
| Ruhr University Bochum | 22 |
| Saarland University (Campus Homburg) | 1 |
| TU Dresden | 18 |
| Technical University of Munich | 5 |
| Ulm University | 2 |
| University of Bonn | 3 |
| University of Cologne | 46 |
| University of Duisburg-Essen | 33 |
| University of Freiburg | 1 |
| University of Göttingen | 51 |
| University of Hamburg | 14 |
| University of Lübeck | 24 |
| University of Münster | 29 |
| University of Rostock | 9 |
| University of Tübingen | 22 |

**S2: Importance of Pharmacology at MHH**
At MHH, the importance of pharmacology is predominantly rated as "high."

**S3: Perceived Importance of Pharmacology at the University of Leipzig**
At the University of Leipzig, the rating “high” also predominates.

**S4: Evaluation of University Teaching at MHH**
At MHH, university teaching is predominantly perceived as “good” and “very good.”

**S5: Evaluation of University Teaching at the University of Leipzig**
At the University of Leipzig, university teaching is mainly rated as “good” or “satisfactory.”

**S6: “Other” Preparation Methods for the University Pharmacology Exam**
Responses regarding alternative materials used to prepare for the university pharmacology exam.

**S7: Other Preparation Methods for the State Examination**
Responses regarding alternative materials used to prepare for the second state examination.

**S8: Other Information on the Use of Textbooks**
As part of the survey, participants could provide further details via a free-text option.

**S9: Use of English Literature – Overall Results**
Use of English-language literature in the overall average.

**S10: Other Information on the Use of English Literature – Overall Results**
Further information on the use of English-language literature based on overall responses.

**S11: Desired Improvements to Textbooks – Other Student Comments**
Suggestions from students regarding potential improvements to textbooks, collected via free-text responses.

**S12: Perceived Confidence in Pharmacology at MHH**
Responses regarding the perceived level of confidence in pharmacology at MHH.

**S13: Perceived Confidence in Pharmacology at the University of Leipzig**
Responses regarding the perceived level of confidence in pharmacology at the University of Leipzig.
